# Lncrna FEZf1-as1 negatively regulates ETNK1 to promote malignant progression of renal cell carcinoma

**DOI:** 10.5937/jomb0-39710

**Published:** 2023-03-15

**Authors:** Jiangyong Lou, Xiaoming Liu, Xiaodong Fan, Xiaoming Xu, Zhichao Wang, Liqun Wang

**Affiliations:** 1 The Second Hospital of Yinzhou, Department of Urology, Ningbo, China; 2 The Second Hospital of Yinzhou, Department of Endocrinology, Ningbo, China

**Keywords:** LncFEZF1-AS1, ETNK1, renal cell carcinoma, malignant progression, LncFEZF1-AS1, ETNK1, karcinom bubrežnih ćelija, maligna progresija

## Abstract

**Background:**

To explore the role of LncFEZF1-AS1 in renal cell carcinoma (RCC) tissues and cells, and the possible molecular mechanism.

**Methods:**

Expressions of LncFEZF1-AS1 in RCC tissues and adjacent ones were detected. The association of LncFEZF1-AS1 level with clinical data of RCC patients was also analyzed. Besides, the differential expressions of LncFEZF1-AS1 in a variety of RCC cell lines were also determined. Then the LncFEZF1-AS1 knockdown model was constructed in RCC cell line to further determine the influences of LncFEZF1-AS1 on the proliferative ability and migration of RCC cells through CCK8 and Transwell experiments. Furthermore, luciferase reporter gene experiment were used to validate the combination of LncFEZF1-AS1 to ETNK1.

**Results:**

Results suggested that expression of LncFEZF1-AS1 was noticeably higher in RCC tumor tissues and the RCC cells. Clinical pathological data analysis also suggested that high LncFEZF1-AS1 expression was in correlation with the pathological stage and the incidence of distant metastasis in RCC patients, and the poor overall survival rate. In vitro experiments demonstrated that knocking down of LncFEZF1-AS1 markedly repressed the proliferation and migration of RCC cell lines. Bioinformatics suggested that LncFEZF1-AS1 can interact with the downstream target gene ETNK1, which was confirmed by the luciferase reporter gene experiments. Western Blot results revealed that knocking down of LncFEZF1-AS1 markedly enhanced ETNK1. qRT-PCR analysis indicated that ETNK1 level was under-expressed in RCC tissues and in negative correlation with LncFEZF1-AS1. Further experiments suggested that knockdown of ETNK1 partially reversed the inhibitory effects of LncFEZF1-AS1 silencing on the proliferative and migrative abilities of RCC cells.

**Conclusions:**

LncFEZF1-AS1 could facilitation the proliferative and migration of RCC cells by regulating the expression of ETNK1. Therefore, FEZF1-AS1 might function as a cancer-promoting factor and possible new therapeutic target for RCC.

## Abstract (sr)

**Uvod:**

Cilj je bio da se istraži uloga ulogu LncFEZF1-AS1 u tkivima i ćelijama karcinoma bubrežnih ćelija (RCC), kao i mogući molekularni mehanizam.

**Metode:**

Detektovane su ekspresije LncFEZF1-AS1 u RCC tkivima i susednim tkivima. Takođe je analizirana povezanost nivoa LncFEZF1-AS1 sa kliničkim podacima pacijenata sa RCC. Pored toga, takođe su određene diferencijalne ekspresije LncFEZF1-AS1 u različitim RCC ćelijskim linijama. Zatim je konstruisan model obaranja LncFEZF1-AS1 u ćelijskoj liniji RCC da bi se dalje odredili uticaji LncFEZF1-AS1 na proliferativnu sposobnost i migraciju RCC ćelija kroz eksperimente CCK8 i Transvell. Štaviše, eksperiment sa reporterskim genom luciferaze je korišćen za validaciju kombinacije LncFEZF1-AS1 sa ETNK1.

**Rezultati:**

Rezultati sugerišu da je ekspresija LncFEZF1-AS1 bila primetno veća u tumorskim tkivima RCC i ćelijama RCC. Analiza kliničkih patoloških podataka takođe je sugerisala da je visoka ekspresija LncFEZF1-AS1 bila u korelaciji sa patološkim stadijumom i incidencom udaljenih metastaza kod pacijenata sa RCC, i lošom stopom ukupnog preživljavanja. In vitro eksperimenti su pokazali da obaranje LncFEZF1-AS1 značajno potiskuje proliferaciju i migraciju RCC ćelijskih linija. Bioinformatika je sugerisala da LncFEZF1-AS1 može da interaguje sa nizvodnim ciljnim genom ETNK1, što je potvrđeno eksperimentima sa reporterskim genom luciferaze. Rezultati Vestern Blota otkrili su da je obaranje LncFEZF1-AS1 značajno poboljšalo ETNK1. kRT-PCR analiza je pokazala da je nivo ETNK1 bio nedovoljno izražen u RCC tkivima i u negativnoj korelaciji sa LncFEZF1-AS1. Dalji eksperimenti su sugerisali da je nokdaun ETNK1 delimično preokrenuo inhibitorne efekte utišavanja LncFEZF1-AS1 na proliferativne i migracione sposobnosti RCC ćelija.

**Zaključak:**

LncFEZF1-AS1 bi mogao olakšati proliferaciju i migraciju RCC ćelija regulacijom ekspresije ETNK1. Stoga, FEZF1-AS1 može funkcionisati kao faktor koji promoviše rak i mogući novi terapeutski cilj za RCC.

## Introduction

Renal cell carcinoma (RCC) is a common type of malignant tumors of the urogenital system, which accounts for a relatively high rate of adult malignant tumors [1] [2] [3]. Moreover, the global incidence of RCC is increasing in recent years, and the mortality rate of RCC has also increased markedly, especially for the elderly aged 60 years and over [1] [2]. Though specific etiology of RCC remains unclear, it has been found that the incidence of RCC has a certain correlation with many different factors [4] [5]. At present, Radiotherapy and chemotherapy are not effective for RCC. Besides, the effect of immunotherapy is poor and some patients cannot tolerate, while the targeted therapy has problems such as drug resistance and complications. The current main treatment for RCC is still the kidney radical surgical resection [6] [7]. However, even after radical surgery, some patients still have the possibility of recurrence and metastasis [8] [9]. Therefore, it is imperative to look deeply for the potential mechanisms of RCC development and discover possible targets that can be used for RCC [10] [11].

At present, the definition of LncRNA is »RNA with original or shear transcript function, does not match the existing concepts of small molecule RNA such as miRNA, piRNA and snoRNA, or cannot be classified as structural RNA« [12] [13]. LncRNA is powerful and has an irreplaceable role in all levels of life metabolism, thus affecting various biological processes [13]. Therefore, lncRNA exerts an extremely important effect in malignant tumors [14] [15] [16] [17].

Previous evidence suggested that LncRNA-FEZF1-AS1 can promote proliferative and metastatic abilities of colorectal cancer by regulating PKM2 signal [18] [19]. However, there is no relevant research on FEZF1-AS1 and RCC in the reported literature. Therefore, we initially explored the levels of Lnc-FEZF1-AS1 in RCC cells through qRT-PCR, and further explored the potential mechanism.

## Materials and methods

### Patients and RCC samples

50 specimens of RCC and their matched normal renal tissue samples adjacent to cancer were stored in the biological resource specimen bank of our hospital at -80°C. The current research was conducted after getting the informed consent of each patient and the hospital ethical committee approval, in accordance with the principles of the Declaration of Helsinki. Each experimental sample included kidney cancer tissue removed after surgery and paired adjacent normal renal tissue (distance from cancer tissue > 2 cm). All samples were diagnosed by at least two pathologists, with complete clinicopathological information and follow-up information.

### Cell lines and reagents

Five human RCC cells (Caki-1, Caki-2, 769-P, 786-O, ACHN) and one normal renal tubular epithelial cell (HK-2) were acquisitioned from ATCC company (Manassas, VA, USA). Cells were cultivated in Dulbecco's Modified Eagle Medium with 10% FBS in an incubator (at 37°C, 5% CO_2_) (Gibco, Rockville, MD, USA).

### Transfection

The negative control vector (sh-NC) and the vector containing the LncFEZF1-AS1 knockdown expression sequence (sh-FEZF1-AS1) were obtained from Shanghai GenePharma Co., Ltd. Cell transfection was done via Lipofetamine 3000 according to instructions.

### Cell proliferation experiments

Transfected cells were transferred to 6-well plates. CCK-8 (Beyotime Biotechnology, Shanghai, China) was added to cells in 6-well plates after 24 h, 48 h, 72 h and 96 h, respectively. After incubated for another 2 hours, then the absorbance of each well was measured under a microplate reader at 490 nm.

### 5-ethynyl-2-deoxyuridine (EdU) assay

The cells were treated with EdU (50 μm) and were then incubated for 2h. AdoLo and DAPI were used for cell staining, followed by being observed using a fluorescent microscope.

### Transwell cell migration and invasion assay

The diluted serum-free medium (5×10^5^) of the cells was seeded on the upper layer of the Matrigel chamber. 700 μL medium with 20% FBS was placed in the bottom chamber. After the transwell was incubated for the specified time, the lower permeabilized cells were collected. Following washed by PBS, penetrating cell counts were performed in 5 random fields.

### Quantitative real-time PCR (qRT-PCR)

Total RNA was extracted using TRIzol (Beyotime, Shanghai, China), and total RNA was Reverse transcribed into cDNA. The primers were listed below (5'-3'): LncFEZF1-AS1: F: GTAACTGGCCTCCC-CAAACG; R: GGTCCGATCGAGTCAAGGTT; ETNK1: F: GCAACCCAGCCATTTTCAGTTTA, R: AAGCA-GAAGCCTTGACCCTC; β-actin: F: CCTGGCAC-CCAGCACAAT, R: GCTGATCCACATCTGCTGGAA.

### Western blot

RIPA+PMSF reagent (Keygen, Nanjing, China) was used for the total protein extraction. In addition, total protein concentration was calculated by Bicinchoninic acid method (Keygen, Nanjing, China). Protein samples were gel separated by Sodium dodecyl-sulfate polyacrylamide gel electrophoresis and transferred to polyvinylidene difluoride membrane and incubated with antibodies against Anti-ETNK1 (Santa Cruz, Santa Cruz, CA, USA).

### Luciferase Dual Assay

Wild-type FEZF1-AS1 and the binding site mutant FEZF1-AS1 were constructed into the pMIR. HEK293T cells were transfected with pcDNA-NC/pcDNA-ETNK1 and pMIR luciferase reporter plasmids. Then the dual luciferase reporter assay QIAGEN (Duesseldorf, Germany) was performed to determine the reporter luciferase activity of cells.

### Statistically analyses

SPSS 22.0 (SPSS Inc., Chicago, IL, USA) was adopted for statistical analyses. Survival was evaluated using Kaplan-Meier. P<0.05 indicates statistically significant difference.

## Results

### LncFEZF1-AS1 was upregulated in RCC tissues and cells

**Figure 1:**
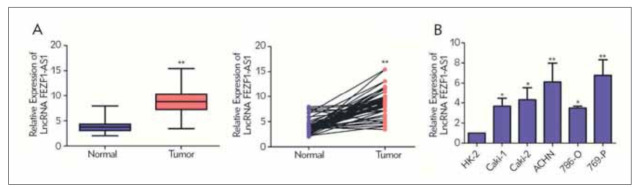
FEZF1-AS1 is highly expressed in RCC tissues and cell lines. (A) qRT-PCR analysis of the FEZF1-AS1 expression in tumor tissues and adjacent tissues of RCC patients; (B) qRT-PCR analysis of the level of FEZF1-AS1 in RCC cell lines. *P<0.05,**P<0.01.

Firstly, we showed that the LncFEZF1-AS1 level in the tumor tissue of RCC patients was markedly increased than that of the adjacent ones (Figure 1A). Besides, compared to HK-2, FEZF1-AS1 was markedly overexpressed in RCC cells, especially in the ACHN and 769-P cell lines (Figure 1B). According to the LncFEZF1-AS1 level in RCC tissues, patients were classified into high LncFEZF1-AS1 expression group and low LncFEZF1-AS1 expression group. We found that RCC patients in high FEZF1-AS1 expression group were more likely to occur distant metastasis (Table 1). At the same time, patients with high FEZF1-AS1 levels had worse overall survival (OS) rates. Therefore, a new biological indicator to predict the malignant progression of RCC may be LNCFEZF1-AS1 in the above results.

**Table 1:** Baseline data of patients were included in this study.

Parameters	Number<br>of cases	LncRNA FEZF1-AS1<br>expression		P-value
		Low (n=30)	High (n=20)	
Age (years)				0.485
<60	22	12	10	22
≥60	28	18	10	28
Gender				0.729
Male	26	15	11	26
Female	24	15	9	24
T stage				0.018
T1-T2	30	22	8	30
T3-T4	20	8	12	20
Lymph node metastasis				0.322
No	34	22	12	34
Yes	16	8	8	16
Distance metastasis				0.033
No	37	24	13	37
Yes	13	4	9	13

### Knockdown of FEZF1AS1 inhibited the proliferative ability and migration in RCC cells

**Figure 2:**
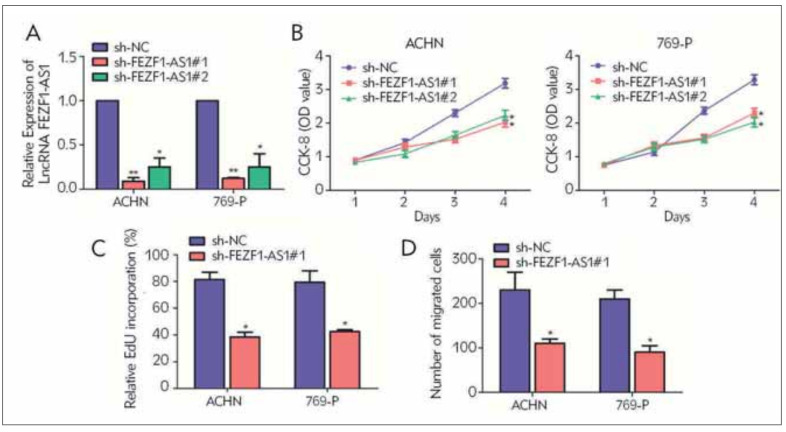
Knocking down FEZF1-AS1 can inhibit the proliferation and migration ability of RCC cells. (A) qRT-PCR verification of the interference efficiency after transfection of FEZF1-AS1 knockdown vectors in RCC cell lines ACHN and 769-P; (B) CCK-8 cell proliferation experiment was used to detect cell proliferation in RCC cell lines ACHN and 769-P after transfection with FEZF1-AS1 knockdown vector; (C) EdU test was used to detect the number of RCC positive proliferating cells after transfection with FEZF1-AS1 knockdown vector in RCC cell lines ACHN and 769-P; (D) Transwell migration was used to detect the cell migration ability after transfection of FEZF1-AS1 knockdown vector in RCC cell lines ACHN and 769-P. *P<0.05, **P<0.01.

To figure out the functional roles of LncFEZF1-AS1 in RCC, the FEZF1-AS1 knockdown vector was first successfully constructed, and then transfected into ACHN and 769-P cells to obtain RCC cell lines with stable FEZF1-AS1 knockdown expression (Figure 2A). Subsequently, the CCK-8 experiment revealed that knocking down LncFEZF1-AS1 markedly inhibited the proliferative ability of the RCC cells (Figure 2B). Similarly, the EdU experiment also indicated that FEZF1-AS1 silencing reduced the number of RCC proliferating cells compared to the sh-NC group, suggesting that the proliferative capacity was inhibited (Figure 2C). Besides, the Transwell migration experimentation showed that compared with the control vector sh-NC, RCC cell migration ability was markedly reduced after knockdown of FEZF1-AS1 (Figure 2D).

### Mutual regulation between FEZF1-AS1 and ETNK1

**Figure 3:**
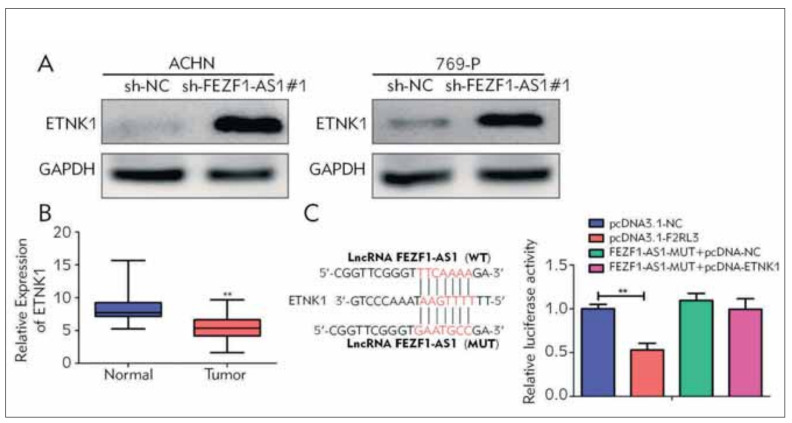
FEZF1-AS1 targets and regulates ETNK1. (A) Western Blot verification of ETNK1 expression level after transfection of FEZF1-AS1 knockdown vector in RCC cell lines ACHN and 769-P; (B) qRT-PCR analysis of ETNK1 expression in RCC tumor tissues and adjacent tissues; (C) The luciferase reporter gene experiment was performed to detect the targeting relationship between FEZF1-AS1 and ETNK1. **P<0.01.

To further explore the regulatory mechanism of FEZF1-AS1 promoting RCC malignant progression, database analysis results suggested that FEZF1-AS1 can interact with downstream target gene ETNK1. Western Blot results indicated that ETNK1 expression was prominently up-regulated after in knockdown of FEZF1-AS1 (Figure 3A). Besides, qRT-PCR analysis also suggested that the ETNK1 level was remarkably reduced in RCC tissues than that of the adjacent ones (Figure 3B). Interestingly, we found that levels of ETNK1 and FEZF1-AS1 exhibited a negative correlation in RCC tissues. Further luciferase reporter gene experiments also suggested that FEZF1-AS1 sequence has a specific binding area for ETNK1 (Figure 3C).

### FEZF1-AS1 inhibited ETNK1 expression in RCC cell lines

**Figure 4:**
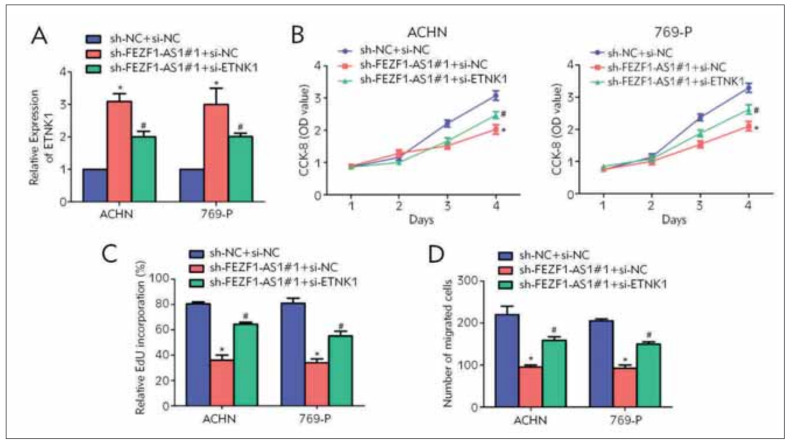
FEZF1-AS1 can regulate ETNK1 to promote the occurrence and development of RCC. (A) qRT-PCR analysis of ETNK1 expression levels after co-transfection of FEZF1-AS1 and ETNK1 vectors in RCC cell lines ACHN and 769-P; (B) CCK-8 cell proliferation experiment was used to detect cell proliferation in RCC cell lines ACHN and 769- P after co-transfection of FEZF1-AS1 and ETNK1 knockdown vectors ; (C) EdU test was used to detect the number of RCC positive proliferating cells after co-transfection of FEZF1-AS1 and ETNK1 knockdown vectors in RCC cell lines ACHN and 769-P; (D) Transwell migration was used to detect the cell migration ability after co-transfection of FEZF1-AS1 and ETNK1 knockdown vectors in RCC cell lines ACHN and 769-P. Data are average ±SD, * represents statistical significance with sh-NC+si-NC, # represents statistical significance with sh-FEZF1-AS1+si-NC, *#P<0.05.

To further asses the mutual regulation mechanism between FEZF1-AS1 and ETNK1 in RCC, ETNK1 was silenced in FEZF1-AS1-knockdown ACHN and 769-P cells. The consequence of qRT-PCR experiments displayed that FEZF1-AS1 knockdown remarkably increased the level of ETNK1; which was further decreased after ETNK1 knockdown (Figure 4A). CCK-8 and EdU experiments both showed that, the RCC cell proliferation was increased after co-transfection of FEZF1-AS1 and ETNK1 knockdown vectors, compared with the single transfection of FEZF1-AS1 knockdown vector (Figure 4B, C), suggesting that knockdown of ETNK1 can reverse the effect of LncFEZF1-AS1 on the proliferative ability of RCC cells. Besides, Transwell migration experiments showed that knockdown of ETNK1 partially reversed the inhibitory effects of LncFEZF1-AS1 silencing on the migrative ability of RCC cells (Figure 4D). These above results showed that LncFEZF1-AS1regulated the malignant progression of RCC via inhibiting the ETNK1 expression.

## Discussion

LncRNA is a RNA molecule that does not encode proteins and has a transcript greater than 200 nucleotides [12] [13]. The human genome contains a large amount of LncRNA, and researches have confirmed that LncRNA exerts significant roles in the development of human tumors [14] [15]. lncRNA are a regulatory factor for gene expression and can achieve regulation of gene expression before and after transcription through multiple pathways. This regulatory mechanism is extremely complex, and it is often achieved by the interaction of multiple LncRNAs and multiple proteins to form a regulatory network to achieve precise regulation of protein expression [13]. Therefore, LncRNAs may become efficient and accurate targets, with broad clinical practical prospects [13] [14] [15]. Here, we mainly explored the level of LncFEZF1-AS1 in RCC and its mechanism in the tumor progression of RCC.

Detection results of lncRNAFEZF1-AS1 level in 50 RCC patients' tumor tissues and their adjacent ones suggested that LncFEZF1-AS1 was markedly increased in RCC tissues than in the adjacent ones; and its expression was in positive correlation with pathological staging, the incidence of distant metastasis as well as the poor prognosis. Therefore, we hypothesized that LncFEZF1-AS1 might be an oncogenic gene in RCC. Subsequent in vitro analysis showed that FEZF1-AS1 knockdown markedly downregulated the proliferative ability and migration of RCC cells, thus inhibiting the progression of RCC.

Through bioinformatics prediction, it was found that FEZF1-AS1 can base pair complementary with the 3'-UTR of ETNK1, thus we speculate that it may affect the expression of ETNK1, thereby promoting the malignant development of RCC. Luciferase reporter gene experiments also confirmed the above speculation. Moreover, knocking down FECF1-AS1 markedly up-regulated ETNK1 in RCC cells. Besides, further findings suggested that FEZF1-AS1 and ETNK1 in RCC tissues suggested a markedly negative correlation. Additionally, to explore the effect of the LncFEZF1-AS1/ETNK1 axis on the progression of RCC, LncFEZF1-AS1 and ETNK1 knockdown vectors were co-transfected into RCC cells. Cell functional results suggested that knockdown of ETNK1 can neutralization the inhibition affect of RCC cell proliferative ability and migration caused by knockdown of LncFEZF1-AS1. The above research findings demonstrated that there may be a feedback loop regulation circuit: FEZF1-AS1 could regulate the ETNK1 expression, thus jointly affecting the malignant progression of RCC.

## Conclusion

In sum, we found that LncFEZF1-AS1 exerts a cancer promoting role in RCC. FEZF1-AS1 can affect the ETNK1 expression to cause malignant progression of RCC.

Our findings may provide potential novel targets for the treatment of RCC.

## Dodatak

### Conflict of interest statement

All the authors declare that they have no conflictof interest in this work.
